# Upregulation of flotillin-1 promotes invasion and metastasis by activating TGF-β signaling in nasopharyngeal carcinoma

**DOI:** 10.18632/oncotarget.6483

**Published:** 2015-12-07

**Authors:** Sumei Cao, Yanmei Cui, Huiming Xiao, Miaoqing Mai, Chanjuan Wang, Shanghang Xie, Jing Yang, Shu Wu, Jun Li, Libing Song, Xiang Guo, Chuyong Lin

**Affiliations:** ^1^ Sun Yat-sen University Cancer Center, State Key Laboratory of Oncology in South China, Collaborative Innovation Center for Cancer Medicine, Guangzhou 510060, China; ^2^ Department of Cancer Prevention Research, Cancer Center, Sun Yat-sen University, Guangzhou, Guangdong 510060, China; ^3^ Zhongshan Ophthalmic Center, Sun Yat-sen University, Guangzhou, Guangdong 510080, China; ^4^ Department of Radiation Oncology, Cancer Center, Sun Yat-sen University, Guangzhou, Guangdong 510060, China; ^5^ Department of the Central Laboratory, The First Affiliated Hospital/School of Clinical Medicine of Guangdong Pharmaceutical University, Guangzhou, Guangdong 510080, China; ^6^ Department of Nasopharyngeal Carcinoma, Cancer Center, Sun Yat-sen University, Guangzhou, Guangdong 510060, China; ^7^ Department of Biochemistry, Zhongshan School of Medicine, Sun Yat-sen University, Guangzhou, Guangdong 510080, China

**Keywords:** nasopharyngeal carcinoma, lymph node metastasis, flotillin-1, TGF-β signaling

## Abstract

Metastasis is the main cause of cancer-related deaths. Nasopharyngeal carcinoma (NPC) is characterized by severe local invasion and high incidence of regional lymph node metastasis, which represents poor prognosis. However, the underlying mechanism that induces lymph node metastasis of NPC remains largely unknown. Herein, we report that flotillin-1 (FLOT1), a component of lipid raft, which was reported to be involved in tumor progression, was robustly upregulated in the NPC samples with lymph node metastasis. High FLOT1 expression was significantly associated with N classification as well as poorer overall and disease-free survivals in 169 archived clinical NPC samples. Overexpression of FLOT1 enhanced the migratory and invasive abilities of NPC cells *in vitro*, and more importantly, promoted invasion into the surrounding tissues and metastasis to lymph nodes *in vivo*. Whereas silencing of endogenous FLOT1 in NPC cells decreased the local invasion and metastasis to lymph nodes. Furthermore, FLOT1 induced the expression and secretion of TGF-β1, facilitated the activation of TGF-β/Smad3 signaling to effectuate epithelial-mesenchymal transition. Our findings present new evidence that FLOT1 plays an important role in promoting aggressive behavior of NPC and provide new insights into the regulatory mechanism of TGF-β signaling.

## INTRODUCTION

Nasopharyngeal carcinoma (NPC) is a highly invasive and metastatic tumor type with high incidence in southern China and Southeast Asia. At the time of initial diagnosis, 90% of patients with NPC show cervical lymph node metastasis, which predicts poor prognosis for this disease [1, 2]. Although recent achievements have been made in the local control of NPC with improved chemoradiotherapy, the paucity of effective treatment strategies, metastasis is the major cause for stagnating survival rate of NPC [3]. Therefore, to better understand the molecular mechanisms which regulate the invasion and metastasis of NPC and to identify valuable molecular biomarkers are essential for prognosis prediction and development of novel therapeutic strategies for NPC metastasis.

Mounting evidence demonstrates the TGF-β signaling pathway is constitutively activated in a wide range of tumor types and is involved in tumor progression by promoting local invasion and even distant metastasis [4–8]. TGF-β signaling is a major inducer of the EMT [9]. When TGF-β signaling is activated, cancer cells acquire access to the EMT program, lose their epithelial characteristics including their polarity and specialized cell-cell contacts, and acquire migratory capacity, allowing them to invade into the surrounding tissues, lymphatic and blood vessels and even remote locations [9–12]. Accordingly, TGF-β signaling-associated induction of the EMT is considered an important step in the progression of tumor metastasis. Conversely, blockade of TGF-β signaling has also been shown to dramatically inhibit invasion and metastasis and has been considered as a therapeutic approach in multiple types of cancer [6, 13–15]. Therefore, further delineation of the mechanisms that regulate TGF-β signaling may provide new clues for the development of targeted cancer therapies.

Flotillin-1(FLOT1), a marker of lipid raft, is recently reported to be overexpressed and correlate with advanced tumor stage as well as poor patient survival in various types of human cancer, including breast, esophageal, liver and lung cancer [16–19]. Notably, high levels of FLOT1 expression were reported to significantly correlate with clinical N (lymph node metastasis) and M (distant metastasis) classification in several human cancers, suggesting that FLOT1 might contribute to cancer metastasis [16–18]. However, the biological role and molecular mechanism of FLOT1 during tumor metastasis remain to be clarified.

Herein, we found that FLOT1 was aberrantly overexpressed in the tumors of NPC patients with lymph node metastasis at diagnosis, and was associated with poor overall and disease-free survivals. Importantly, we demonstrated that FLOT1 promoted the invasion and lymph node metastasis of NPC cells *in vitro* and *in vivo* by activating TGF-β signaling. Taken together, these findings demonstrate that FLOT1 functions as an important regulator of lymph node metastasis by conferring constitutive activation of the TGF-β signaling pathway in NPC.

## RESULTS

### Upregulation of FLOT1 correlates with lymph node metastasis in human NPC

**Figure 1:**
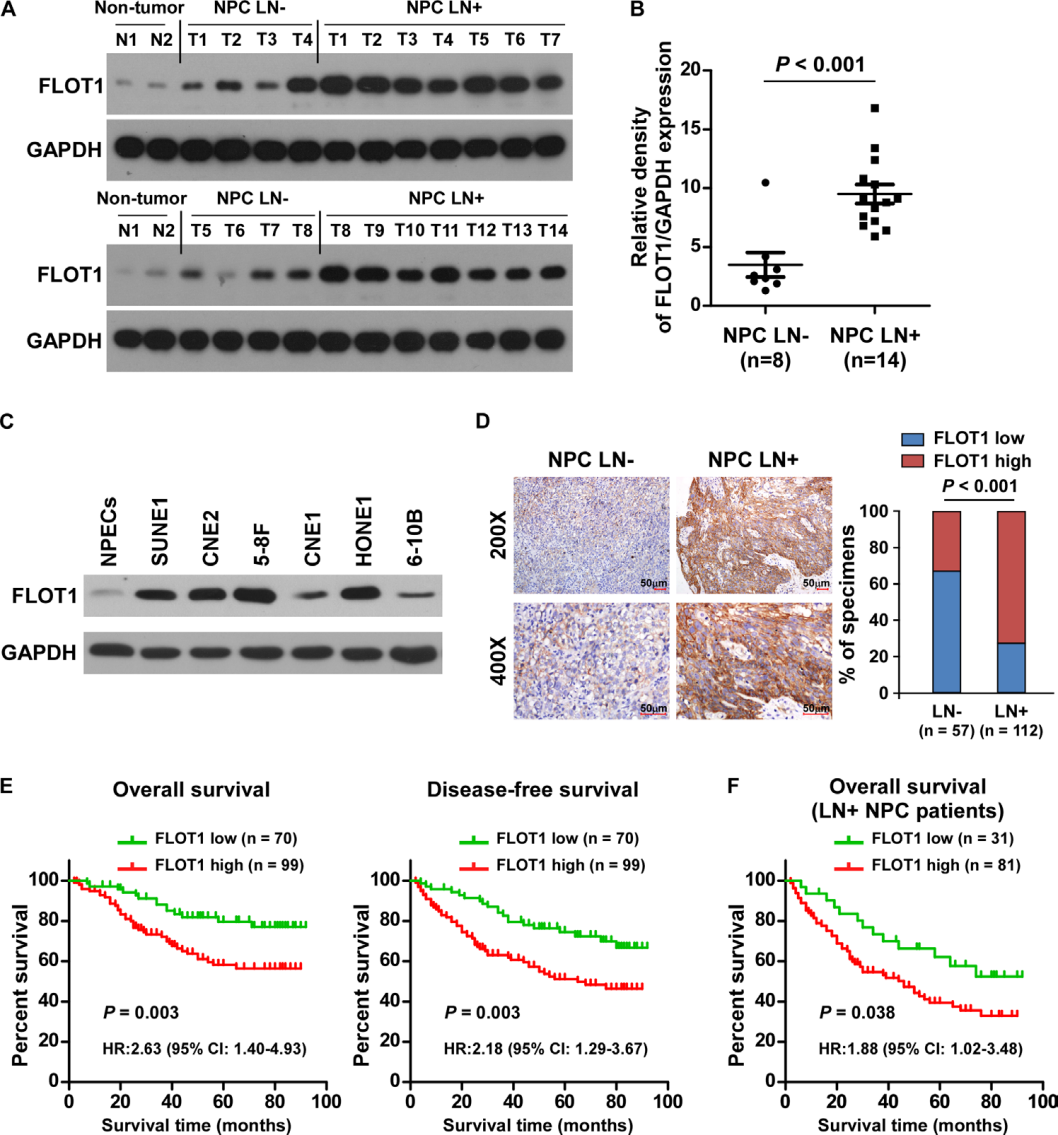
Upregulation of FLOT1 correlates with lymph node metastasis in human NPC **(A)** Western blotting analyses of FLOT1 expression in two non-cancerous nasopharyngeal tissues and tumor tissues from patients with NPC with (LN+, *n* = 14) and without (LN−, *n* = 8) lymph node metastasis. GAPDH was used as a loading control. **(B)** Quantification of western blotting analysis of FLOT1 expression; the average ratio of FLOT1/GAPDH in the two non-cancerous nasopharyngeal tissues was considered as 1.0. Error bars represent mean ± SEM. *P* < 0.001, Student's two-tailed *t*-test. **(C)** Western blotting analyses of FLOT1 expression in 6 cultured NPC cell lines compared with normal nasopharyngeal epithelial cells (NPECs). **(D)** Representative photomicrographs (left) and the percentages (right) of specimens with high and low levels of FLOT1 expression in tumor tissues from patients with NPC with (LN+, *n* = 112) or without (LN−, *n* = 57) lymph node metastasis. Scale bars: 50 μm. Right panel: The Chi-square test was used for the correlation analysis. **(E)** Kaplan–Meier overall survival and disease-free survival curves for patients with NPC stratified by low and high levels of FLOT1 expression (*n* = 169; log-rank test). **(F)** Correlation of FLOT1 expression with overall survival in the subgroup of patients with lymph node metastasis (*n* = 112; log-rank test).

To investigate the role of FLOT1 in the progression of NPC, we first examined FLOT1 expression in NPC cell lines and tissues. Western blotting analysis revealed that FLOT1 was markedly overexpressed in 22 primary NPC tissues compared with non-cancerous nasopharyngeal tissues, and in 6 tested NPC cell lines as compared with normal nasopharyngeal epithelial cells (NPECs) (Figure 1A–1C). Importantly, FLOT1 expression levels were more robustly elevated in NPC tissues from patients with lymph node metastasis (LN+) than the LN− cases (Figure 1A and 1B), suggesting that FLOT1 might play a role in lymph node metastasis in NPC.

We further assessed whether FLOT1 expression was clinically correlated with lymph node metastasis in 169 paraffin-embedded, archived NPC tissues, including 57 lymph node metastasis-negative (LN−) cases and 112 lymph node metastasis-positive cases (LN+; Supplementary Table 1). Higher levels of FLOT1 expression were observed in the tumors of LN+ than LN− cases (Figure 1D). Chi-square test revealed that FLOT1 expression was significantly associated with the lymph node metastasis status (*P* < 0.001) (Figure 1D), suggesting that overexpression of FLOT1 is clinically associated with lymph node metastasis in NPC.

Moreover, FLOT1 was closely associated with patient survival. Patients with high FLOT1 better simulation of tumor invasion survival (*P* = 0.003; hazard ratio (HR) (95% confidence interval (CI)) = 2.63 (1.40–4.93)) and disease-free survival (*P* = 0.003; HR (95% CI) = 2.18 (1.29–3.67)) compared to patients with low FLOT1 expression (Figure 1E). High levels of FLOT1 expression also predicted poorer overall survival (*P* = 0.038; HR (95% CI) = 1.88 (1.02–3.48)) in the subgroup of patients with lymph node metastasis (Figure 1F). In addition, univariate and multivariate analyses revealed that N classification and FLOT1 expression were each recognized as independent prognostic factors in NPC (both *P* < 0.05; Supplementary Table 2 and 3), suggesting that FLOT1 has potential clinical value as a predictive biomarker for disease outcome in NPC.

### FLOT1 enhances the migration and invasion of NPC cells

**Figure 2:**
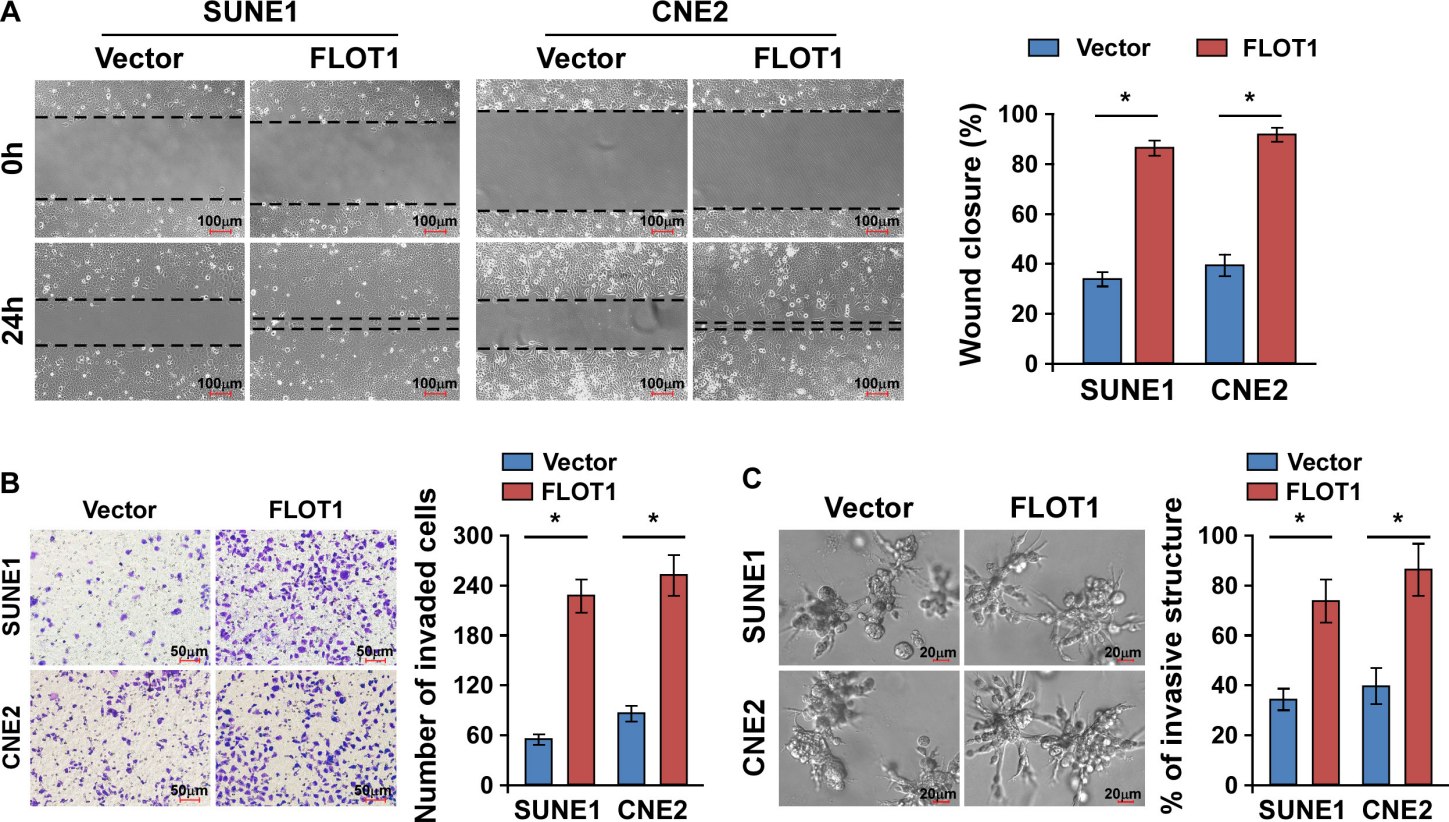
FLOT1 enhances the migration and invasion of NPC cells **(A)** Representative micrographs and quantification of the motility of FLOT1-overexpressing NPC cells in the wound healing assay at 0 h and 24 h compared to vector control cells. Scale bars: 100 μm. **(B)** Representative micrographs and quantification of the invasiveness of FLOT1-overexpressing cells in the transwell matrix invasion assay compared to vector control cells. Scale bars: 50 μm. **(C)** Representative micrographs (left) and quantification of invasive structures (right) of FLOT1-transduced and vector-transduced SUNE1 and CNE2 cells cultured in the 3–D spheroid invasion assay. Scale bars: 20 μm. Error bars represent mean ± SD from three independent experiments, *P < 0.05.

Since acquisition of a migratory and invasive phenotype is necessary for cancer cell dissemination, we next examined whether FLOT1 regulated NPC cell migration and invasion. The NPC cell lines SUNE1 and CNE2 with high invasive capabilities were engineered to exogenously overexpress FLOT1, or silence endogenous FLOT1 expression (Supplementary Figure 1A and 1B). The wound healing assay and transwell matrix penetration assay revealed that overexpression of FLOT1 enhanced the migratory and invasive abilities of NPC cells compared to the respective control cells (Figure 2A and Figure 2B). However, we did not observe a significant increase of cell numbers by FLOT1 overexpression within 24 h (Supplementary Figure 2A). Moreover, a three-dimensional spheroid invasion assay, which is considered to be a better simulation of tumor invasion *in vivo*, revealed that FLOT1-overexpressing cells exhibited active invasive behaviors, characterized by the formation of outward projections from individual cells (Figure 2C). Thus, these results indicate that overexpression of FLOT1 enhances the migration and invasion of NPC cells.

### FLOT1 promotes NPC cell invasion and lymph node metastasis *in vivo*

**Figure 3:**
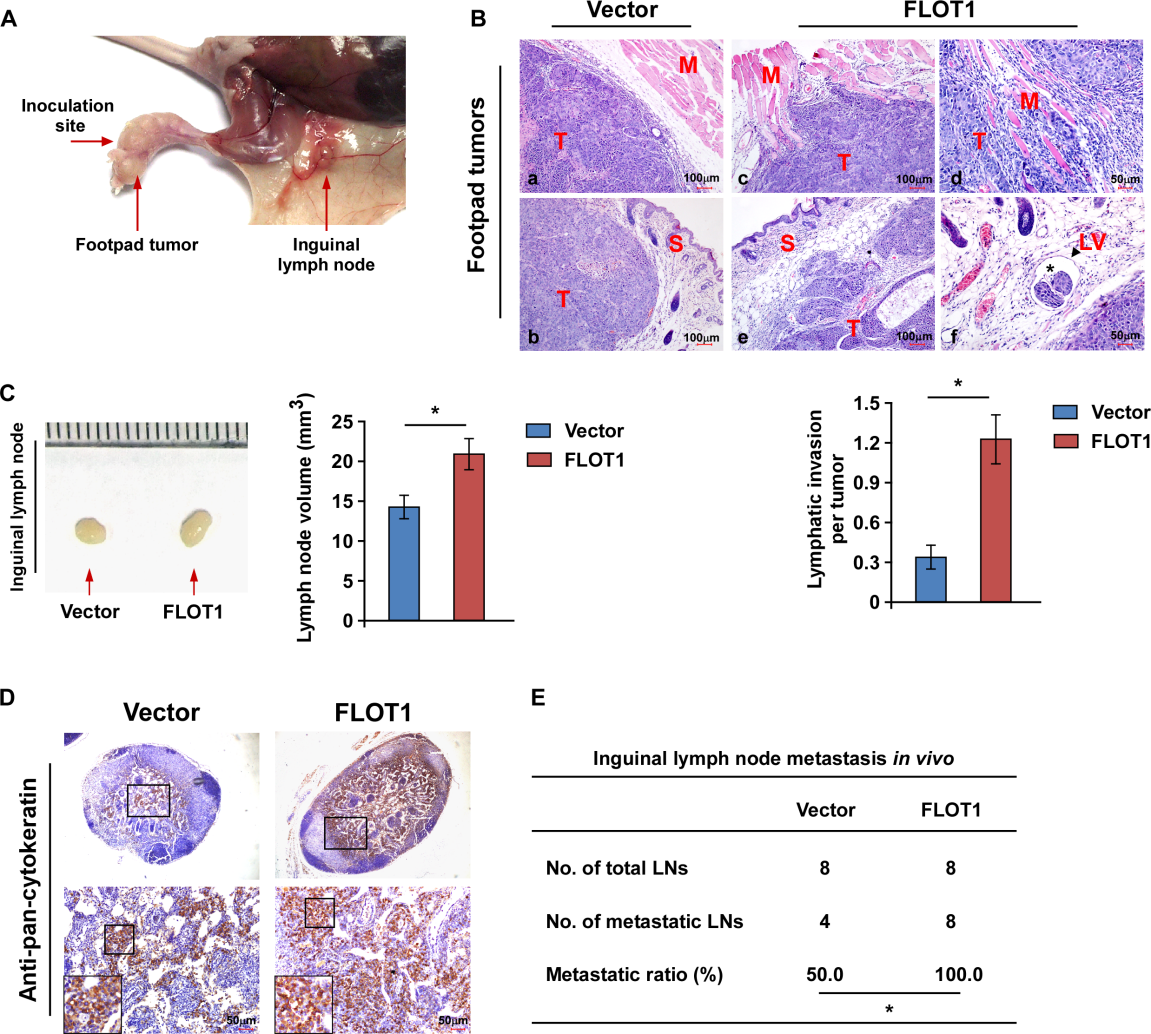
FLOT1 promotes NPC cell invasion and lymph node metastasis *in vivo* **(A)** An inguinal lymph node metastasis model was established by inoculating the foot pads of nude mice with CNE2 cells. **(B)** Upper panel: H & E staining revealed the tumors formed by FLOT1-overexpressing CNE2 cells (c, d, e and f) exhibited a more aggressive phenotype than the vector control cells (a and b), with the FLOT1-overexpressing tumor cells invading into the muscles (c and d), and towards the skin (e), and even into lymphatic vessels (f; asterisk represents tumor cells; arrowheads represent lymphatic vessel). T: tumor; S: skin; M: muscle; LV: lymphatic vessel. Lower panel: Quantification of the amount of peritumoral lymphatic invasion in histologic sections of the tumors formed by FLOT1-overexpressing and vector control CNE2 cells. **(C)** Representative micrographs (left) and volumes of the inguinal lymph nodes (right). **(D)** Representative micrographs of the inguinal lymph nodes immunostained with an anti-pan-cytokeratin antibody. Scale bars: 50 μm. **(E)** Ratios of metastatic inguinal lymph nodes to the total number of inguinal lymph nodes dissected in mice inoculated with the indicated cells, the Chi-square test was used for statistical analysis. Error bars represent mean ± SD; *P < 0.05.

The effects of FLOT1 on cell invasion and lymph node metastasis in NPC were further investigated *in vivo* using the inguinal lymph node metastasis model. Vector-transduced or FLOT1-overexpressing CNE2 cells were inoculated into the foot pads of nude mice the surrounding tissues and (*n* = 8/group; Figure 3A). The resulting foot-pad tumors and inguinal lymph nodes were excised after 4 weeks and analyzed. Histological examination of the primary tumors using H & E staining revealed that the tumors formed by FLOT1-overexpressing CNE2 cells exhibited a more aggressive phenotype, with the FLOT1-overexpressing tumor cells invading towards the muscles, skin and even into the lymphatic vessels, in contrast to the control tumor which showed sharp edges that expanded as spheroids (Figure 3B). Additionally, we found that the lymph nodes in tumors formed from FLOT1-transduced cells had larger volumes and displayed higher numbers of pan-cytokeratin-positive tumor cells than the lymph nodes of the animals injected with the vector-control cells (Figure 3C and 3D). Strikingly, the ratio of metastatic inguinal lymph nodes to the total number of inguinal lymph nodes dissected was markedly higher in the CNE2-FLOT1 group (100.0%, 8/8) than the vector-control group (50.0%, 4/8; Figure 3E). Taken together, these results indicate that FLOT1 promotes invasion and lymph node metastasis in NPC *in vivo*.

### Downregulation of FLOT1 suppresses the aggressive phenotype of NPC cells

**Figure 4:**
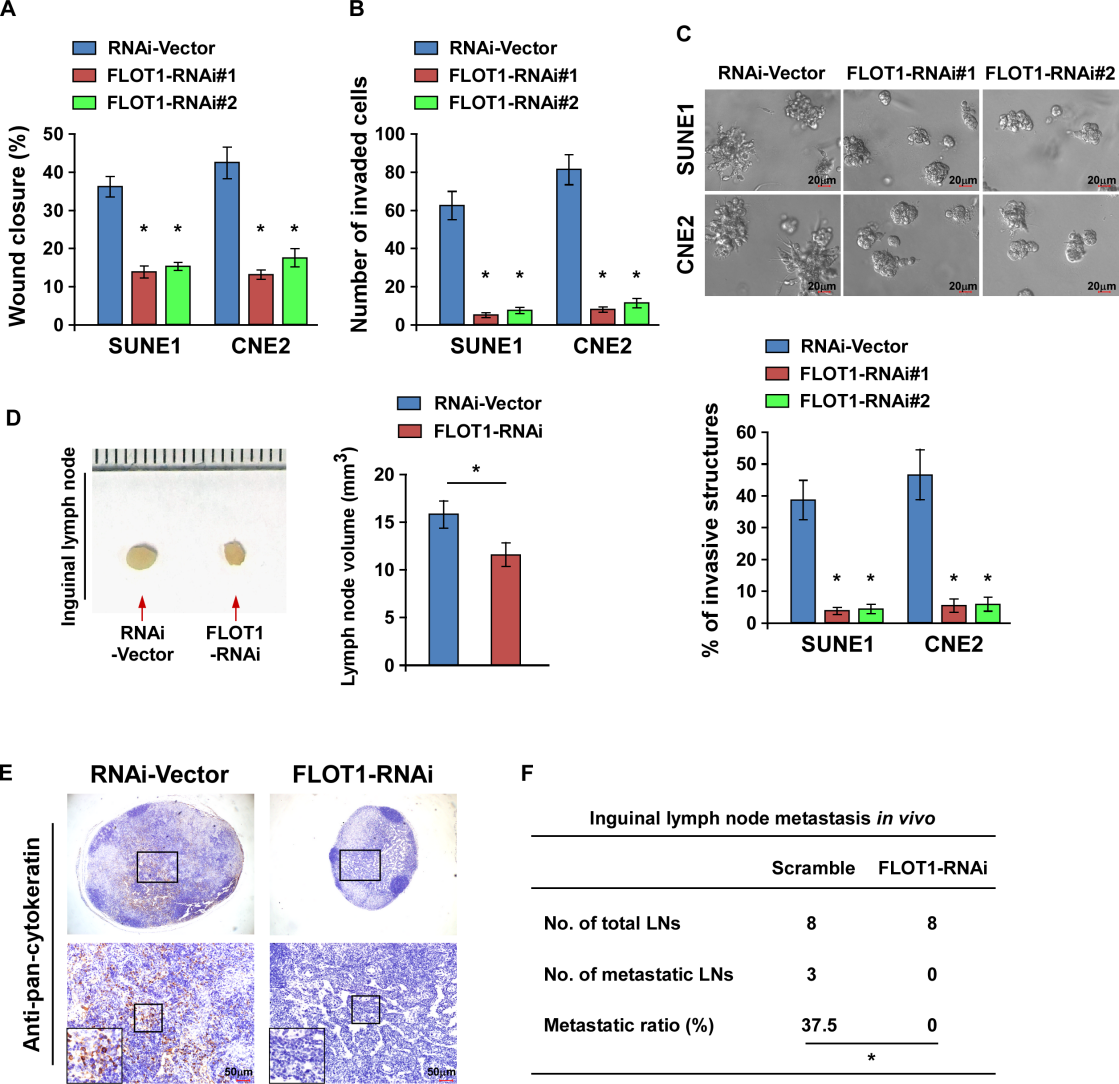
Downregulation of FLOT1 suppresses the aggressiveness of NPC cells **(A and B)** Wound healing assay (A) and transwell matrix invasion assay (B) showing that silencing of FLOT1 reduced NPC cell migration and invasion. **(C)** Representative micrographs (upper) and quantification analysis (lower) of the indicated cells cultured in the 3-D spheroid invasion assay. Scale bars: 20 μm. **(D)** Representative micrographs (left panel) and the volumes of inguinal lymph nodes in mice inoculated with the indicated cells in the inguinal lymph node metastasis model (right panel). **(E)** Representative micrographs of inguinal lymph nodes immunostained with an anti-pan-cytokeratin antibody. Scale bars: 50 μm. **(F)** Ratios of metastatic inguinal lymph nodes to the total number of inguinal lymph nodes dissected in mice inoculated with the indicated cells, the Chi-square test was used for statistical analysis. Error bars represent mean ± SD from three independent experiments; *P < 0.05.

Conversely, the biological role of FLOT1 in NPC metastasis was further confirmed by silencing of FLOT1. Downregulation of FLOT1 significantly reduced the migration and invasion of both SUNE1 and CNE2 cell lines in the wound healing assay, transwell matrix penetration assay and 3–D spheroid formation assay, but not cell proliferation within 24 h (Figure 4A–4C and Supplementary Figure 2B). Moreover, the volume of the lymph nodes, numbers of pan-cytokeratin-positive tumor cells and ratio of metastatic to total inguinal lymph nodes dissected were significantly reduced by knocking down of FLOT1 (Figure 4D–4F). Taken together, these results suggest that downregulation of FLOT1 suppresses the motility of NPC cells *in vitro* and attenuates lymph node metastasis *in vivo*.

### FLOT1 activates the TGF-β signaling pathway

**Figure 5:**
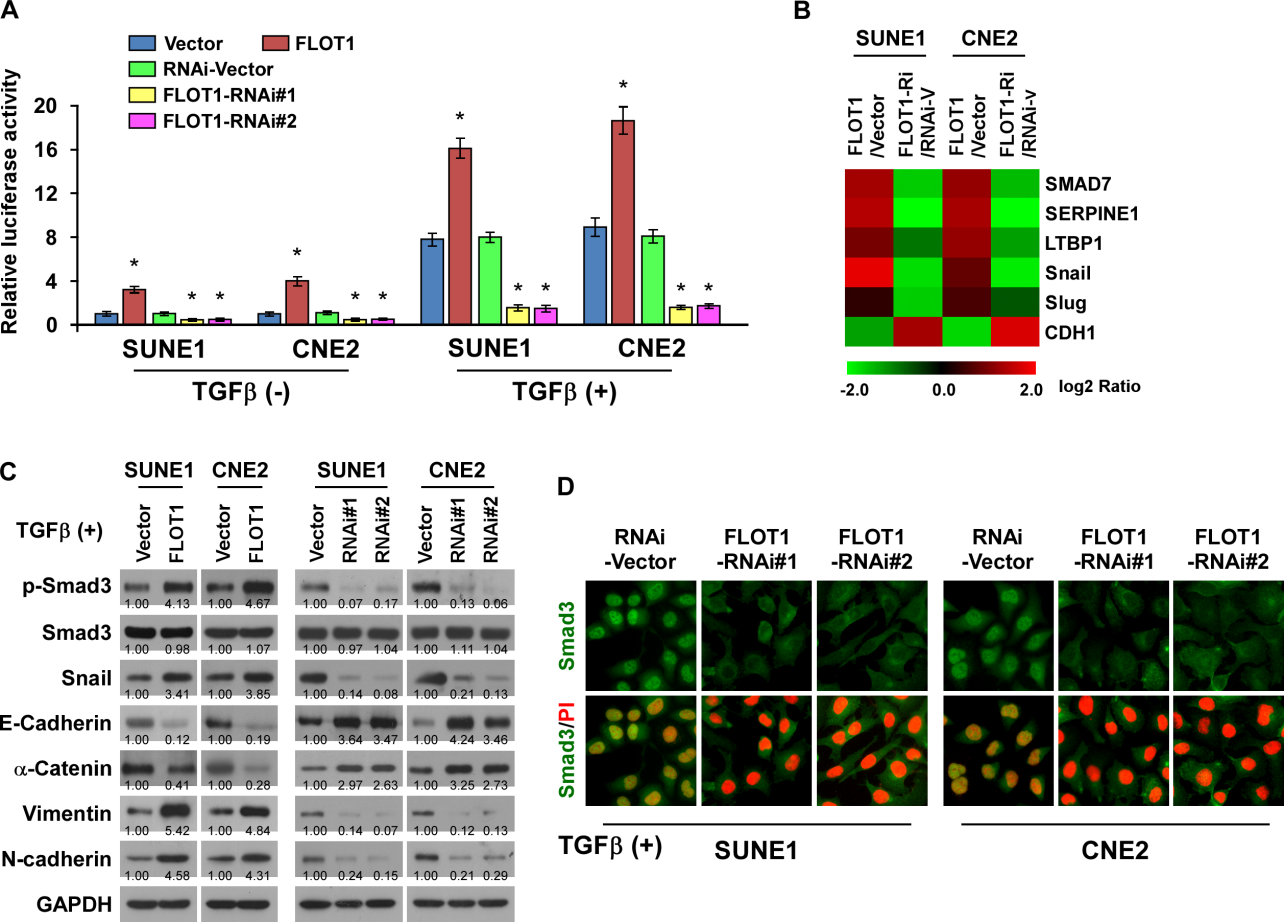
FLOT1 activates the TGF-β signaling pathway **(A)** TGF-β-responsive luciferase activity was measured in the indicated cells after 48 h culture with or without TGF-β1 (5 ng/ml) for 20 h using the dual luciferase assay. Values are mean ± SD of triplicate samples. **(B)** Real-time PCR analysis revealed that FLOT1 regulates the expression levels of numerous well-known genes downstream of TGF-β. The pseudocolors represent the intensity scale of expression in FLOT1- *vs*. vector-transduced cells or FLOT1-RNAi- *vs*. RNAi-vector-transduced cells generated by log_2_ transformation. **(C)** Western blot analysis of the phosphorylated and total levels of Smad3, Snail and expression of EMT markers including E-cadherin, α-catenin, N-cadherin and vimentin in the indicated SUNE1 and CNE2 cells treated with TGF-β1 (5 ng/ml). GAPDH was used as a loading control. **(D)** Subcellular localization of Smad3 in the indicated cells treated with TGF-β1 (5 ng/ml). Error bars represent mean ± SD from three independent experiments; *P < 0.05.

We next investigated whether FLOT1 promoted cell invasion and lymph node metastasis in NPC by activating TGF-β signaling pathway. As shown in Figure 5A–5D, we found that overexpressing FLOT1 enhanced, whereas silencing of FLOT1 reduced, TGF-β responsive luciferase activity, the expression of numerous well-characterized downstream genes of TGF-β signaling and the levels of phosphorylated-Smad3 (an indicator of TGF-β activation), snail, as well as the nuclear translocation of Smad3 in NPC cells. Moreover, overexpression of FLOT1 increased, while silencing FLOT1 reduced the binding capability of Smad3 with SERPINE1 promoter (Supplementary Figure 3). Taken together, these results suggest that FLOT1 activates the TGF-β signaling pathway in NPC cells.

Importantly, we observed that TGF-β-induced luciferase activity and the EMT phenotype were robustly elevated in FLOT1-overexpressing NPC cells, but abrogated by silencing of FLOT1 (Figure 5A, 5C, 5D and Supplementary Figure 4A, 4B), suggesting that FLOT1 plays an important role in sensitizing NPC cells to EMT-inducing signals such as TGF-β.

### FLOT1 induces an autocrine secretion of TGF-β1 in NPC cells

**Figure 6:**
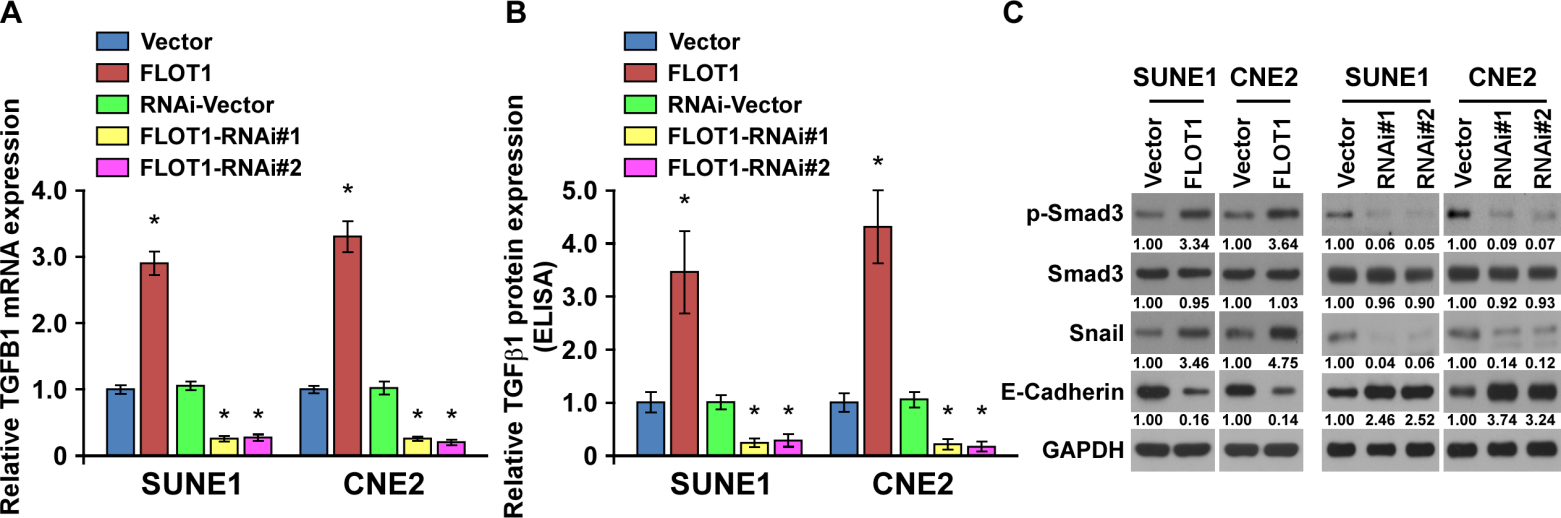
FLOT1 induces an autocrine secretion of TGF-β1 in NPC cells **(A)** Real-time PCR analysis of TGFB1 mRNA expression in vector control cells and FLOT1-transduced NPC cells, or RNAi-vector control cells and FLOT1-silenced NPC cells. Expression levels were normalized to GAPDH. **(B)** TGF-β1 protein levels in the supernatants of the indicated cells were assessed using an enzyme-linked immunosorbent assay. **(C)** Western blot analysis of the phosphorylated and total levels of Smad3, Snail and E-cadherin in the indicated SUNE1 and CNE2 cells. GAPDH was used as a loading control.

Notably, we found that FLOT1 overexpression was able to activated TGF-β signaling in the absence of TGF-β1 treatment. We further found that FLOT1 overexpression facilitated the expression and secretion of TGF-β1, leading to upregulation of Smad3 phosphorlation and Snail expression, and downregulation of E-cadherin in SUNE1 and CNE2 cells (Figure 6A–6C). In contrast, silencing of FLOT1 repressed the expression and secretion of TGF-β1, and activity of TGF-β/Smad3 signaling module (Figure 6A–6C). Thus, our findings revealed that FLOT1 was able to activate the TGF-β signaling by inducing an autocrine secretion of TGF-β1.

### TGF-β signaling activation is required for FLOT1-induced lymph node metastasis

**Figure 7:**
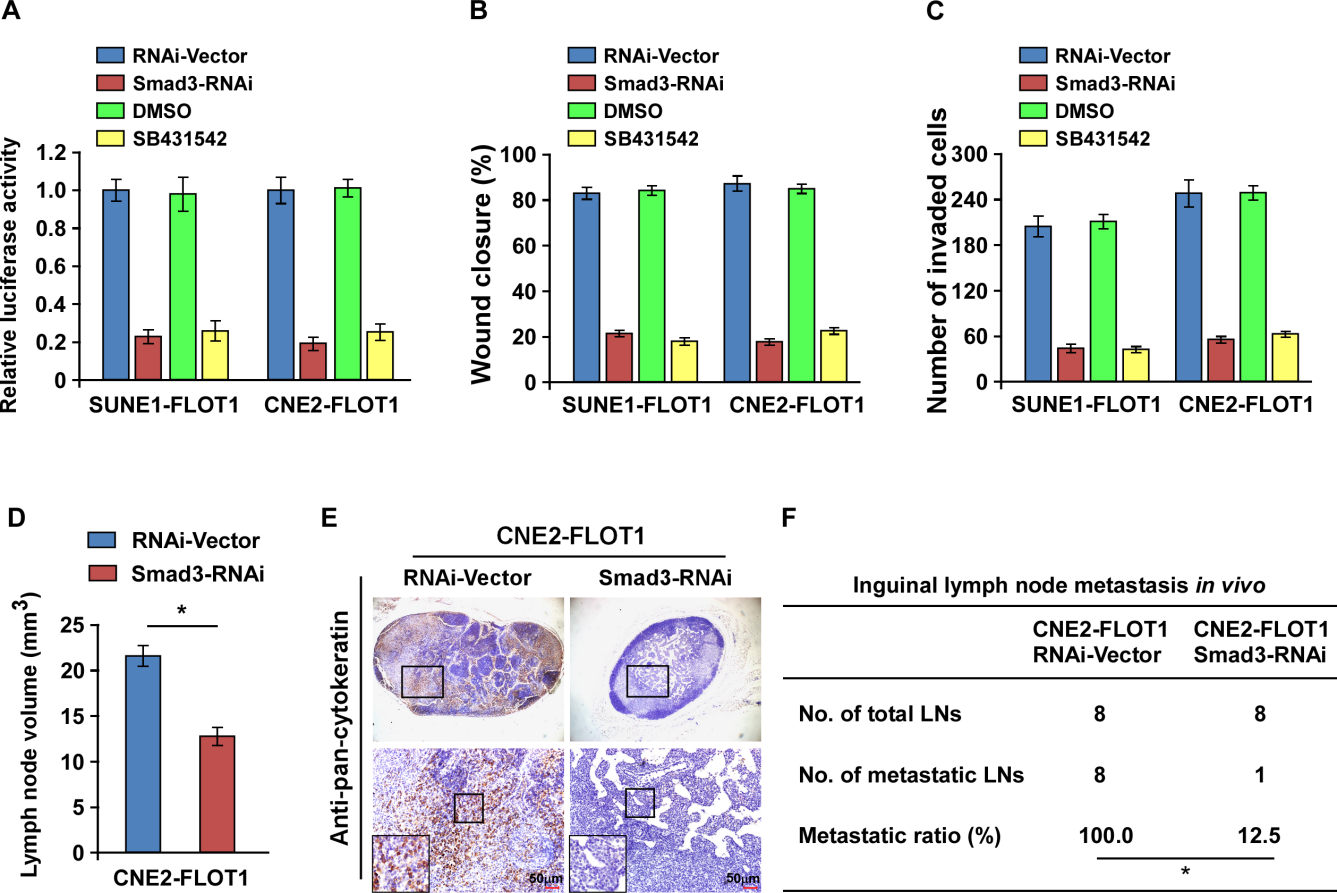
TGF-β signaling activation is required for FLOT1-induced lymph node metastasis in NPC **(A)** TGF-β responsive luciferase activity induced by FLOT1 is suppressed by depletion of Smad3 or treatment with TGF-β inhibitor SB431542 (10 μM) for 24 h in indicated cells. **(B and C)** Promotion of NPC cell migration (B) and invasion (C) induced by FLOT1 is inhibited by Smad3 silencing or SB431542 treatment (10 μM) for 24 h as indicated by wound healing and transwell matrix penetration assays. **(D)** The average volumes of inguinal lymph nodes in each group. **(E)** Representative micrographs of the inguinal lymph nodes immunostained with an anti-pan-cytokeratin antibody. Scale bars: 50 μm. **(F)** The ratios of metastatic to total dissected inguinal lymph nodes from mice inoculated with the indicated cells, the Chi-square test was used for statistical analysis. Error bars represent mean ± SD; *P < 0.05.

We next assessed the functional significance of TGF-β signaling activation in FLOT1-mediated NPC cell metastasis by blocking TGF-β signaling in FLOT1-overexpressing cells via silencing of Smad3 or treating the cells with the TGF-β inhibitor SB431542. As expected, the stimulatory effect of FLOT1 on TGF-β signaling activation was inhibited by silencing of Smad3 or treatment with SB431542 (Figure 7A). Moreover, silencing of Smad3 and treatment with SB431542 both abrogated the effects of FLOT1 on NPC cell migration and invasion, as indicated by the wound healing assay and transwell matrix invasion assay (Figure 7B and 7C). *In vivo*, silencing of Smad3 dramatically reduced both the volume and number of pan-cytokeratin-positive tumor cells detected in the lymph nodes of the inoculated mice (Figure 7D and 7E). In addition, the metastatic lymph node ratio markedly reduced from 100.0% (8/8) to 12.5% (1/8) in the CNE2/FLOT1/RNAi-Vector and CNE2/FLOT1/Smad3 RNAi groups, respectively (Figure 7F). Taken together, these results demonstrate that TGF-β signaling activation is essential for FLOT1-induced invasion and lymph node metastasis in NPC.

### FLOT1 promoted lymph node metastasis independent of FLOT2 in NPC

Previous studies identify that flotillins (FLOT1 and FLOT2) tend to form oligomers and are highly dependent on each other in their expression [20–22]. Notably, FLOT1 depletion typically results in only a moderate or even no depletion of FLOT2 [22, 23]. We then examined whether FLOT1 regulated FLOT2 expression in NPC. As shown in Supplementary Figure 5A, only slight changes of FLOT2 expression were observed in the FLOT1-overexpressing and FLOT1-silencing SUNE1 and CNE2 cells. Moreover, we found that FLOT1 expression was not significantly correlated with FLOT2 expression in ten freshly collected human NPC samples (*r* = −0.174, *P* = 0.769; Supplementary Figure 5B). In addition, using the inguinal lymph node metastasis model, we found that silencing of FLOT2 had no significant effect on the promotion role of FLOT1 in NPC lymph node metastasis *in vivo* (*P* > 0.05; Supplementary Figure 6A–6C), suggesting that FLOT1 promoted lymph node metastasis independent of FLOT2 in NPC.

### Clinical relevance of FLOT1-induced TGF-β signaling activation in NPC

**Figure 8:**
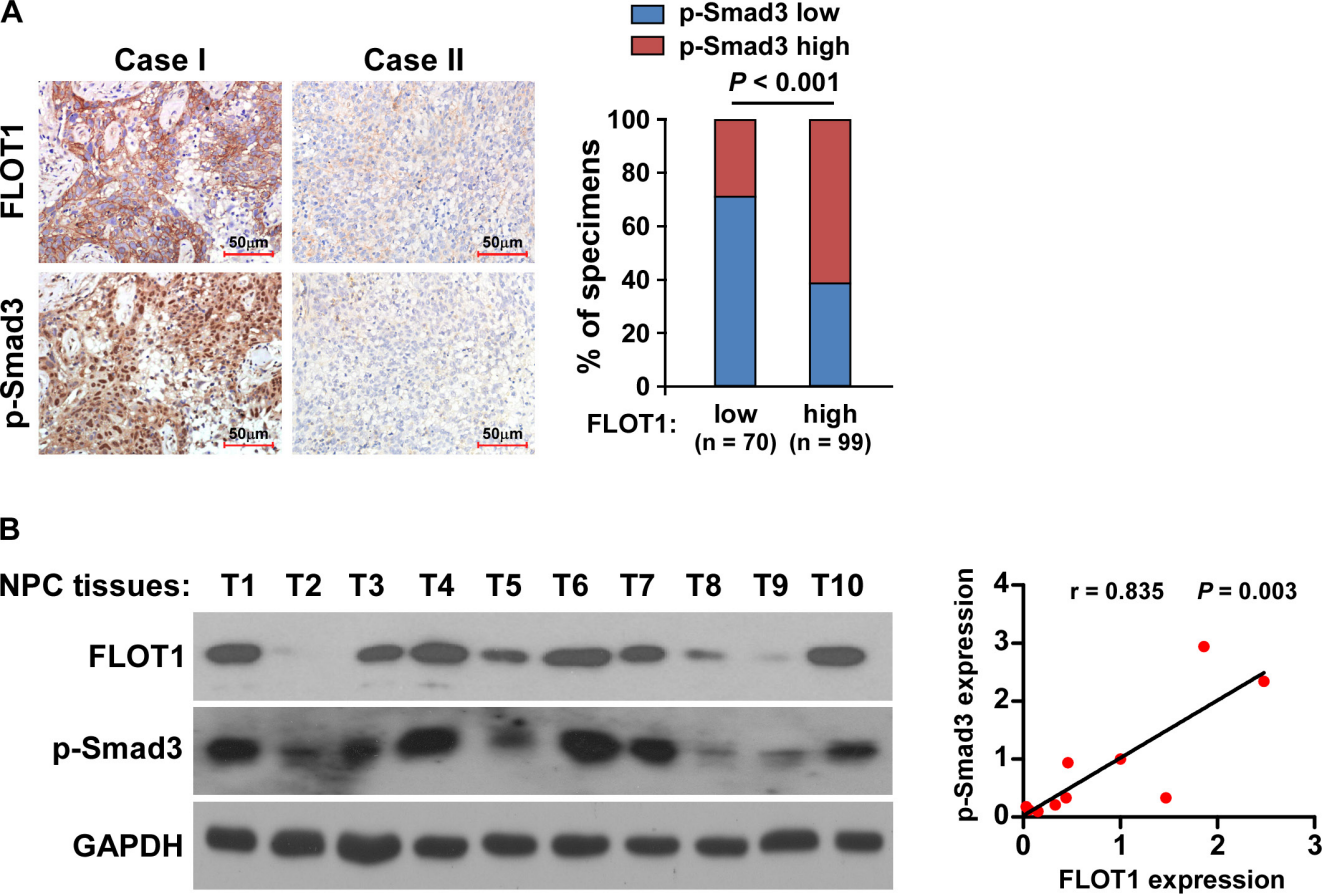
Clinical relevance of FLOT1-induced TGF-β signaling activation in NPC **(A)** FLOT1 expression levels associated with p-Smad3 expression in 169 primary human NPC specimens. Left panel: Two representative cases are shown. Scale bars: 50 μm. Right panel: Percentages of samples showing low or high FLOT1 expression in 169 primary human NPC specimens relative to the levels of p-Smad3. **(B)** Expression analysis (left panel) and correlation (right panel) of FLOT1 expression and p-Smad3 protein expression in ten freshly collected human NPC tissue samples (T). The ratio of sample T1 (FLOT1/GAPDH and p-Smad3/GAPDH) was considered as 1.0. GAPDH was used as a loading control.

Finally, we examined whether the FLOT1/TGF-β signaling pathway axis identified in our study had clinical relevance in human NPC samples. As shown in Figure 8A, IHC analysis revealed that the levels of FLOT1 expression in the 169 human NPC specimens positively correlated with the levels of p-Smad3 (*P* < 0.001; Figure 8A). Moreover, in the ten freshly collected human NPC samples, FLOT1 expression was also strongly associated with the levels of p-Smad3 (*r* = 0.835, *P* = 0.003; Figure 8B). Collectively, these results further support the notion that overexpression of FLOT1 activates the TGF-β signaling pathway and thereby promotes invasion and lymph node metastasis in NPC.

## DISCUSSION

NPC is an extremely endemic malignant tumor type that has a high incidence in southern China. Due to the well-developed lymphatic network in the nasopharynx, NPC has a high incidence of cervical lymph node metastasis at diagnosis, which is associated with a poor prognosis [24, 25]. Hence, there is an urgent need to identify the key molecular alterations that contribute to local invasion and lymph node metastasis in order to provide effective targets for anti-metastatic therapy in NPC. In this study, we found that high expression of FLOT1 significantly correlated with lymph node metastasis and poor survival in human NPC. Moreover, FLOT1 promoted NPC cells to invade into the surrounding tissues and metastasize to lymph nodes *in* vivo. Thus, this study provides new insight into the mechanisms that regulate invasion and metastasis in NPC and suggests the potential of FLOT1 as target for anti-metastatic therapy in NPC.

The TGF-β/Smad pathway is crucial in EMT induction due to its multiple downstream effectors, including Snail, Slug, Twist and ZEB1, which are capable of repressing E-cadherin and subsequently enabling upregulation of mesenchymal promoters [26–28]. Loss of E-cadherin expression initiates a series of signaling events and major cytoskeletal reorganization that promotes the migration and invasion of cancer cells [11, 29]. Consistently, hyperactivation of TGF-β signaling and low levels of E-cadherin were observed in NPC specimens, and were associated with lymph node metastasis status of patients, suggesting that TGF-β signaling might play an important role in the lymph node metastasis in NPC [5, 8, 30, 31]. Herein, we demonstrate that FLOT1 activates TGF-β signaling in NPC cells, leading to upregulation of multiple downstream genes including *Snail* and *Slug*, and downregulation of E-cadherin. Of note, upregulation of FLOT1 significantly correlated with high Smad3 activity and low E-cadherin expression in the human NPC samples, further supporting the notion that aberrant expression of FLOT1 is responsible for the hyperactivation of TGF-β signaling and high metastatic capability of NPC cells. Thus, our findings not only confirm that activation of TGF-β signaling contributes to the malignant behavior of NPC, but also reveal a novel mechanism for activation of TGF-β involving FLOT1 in NPC.

Over the past decades, studies have demonstrated that certain heterotypic signals that originate in the reactive stroma of primary tumor, impinge on neoplastic cells located at the outer edges, and induce these cells to undergo an EMT [32]. Apart from TGF-β, a variety of other EMT-inducing factors, including tumor necrosis factor-α (TNF-α), EGF (epidermal growth factor), HGF (hepatocyte growth factor), and IGF-l (insulin-like growth factor-l), have been implicated [33–36]. Interestingly, FLOT1 has been reported to be crucial for coordinated assembly of signaling complexes and signal transduction induced by TNF-α, EGF, HGF and IGF-l, suggesting that FLOT1 might play an important role in mediating the heterotypic signals originate in the tumor microenvironment [17, 23, 37, 38]. In this study, we also found that the TGF-β-induced signaling activity and EMT phenotype were robustly elevated in FLOT1-overexpressing NPC cells, but abrogated by silencing of FLOT1, suggesting that FLOT1 expression plays an important role in sensitizing NPC cells to TGF-β. Thus, such a FLOT1-mediated mechanism may enhance the heterotypic signals induced by extrinsic stimuli to induce the acquisition of mesenchymal traits by cancer cells and promote metastasis during the late stages of tumor progression.

Notably, the recent study by Zhao L et al. identified that FLOT2, the “sister protein” of FLOT1, was upregulated in NPC and associated with lymph node metastasis of patients [39]. Thus, these results further suggest that flotillins are involved in NPC lymph node metastasis. Importantly, we were able to demonstrate that FLOT1 promoted NPC cells invasion into the surrounding tissues and metastasis to lymph nodes *in vivo* by using the inguinal lymph node metastasis model. Zhao L and colleagues then found that FLOT2 was essential for the TGF-β induced Src signaling activation to effectuate EMT [39]. Instead, we found that FLOT1 promoted EMT by activating the TGF-β/Smad3 signaling module, suggesting that flotillins might activate different signaling modules downstream of TGF-β. Interestingly, we found that FLOT1 was not only required for TGF-β induced EMT but also they can, by themselves when overexpressed, activated TGF-β/Smad3 signaling and promoted NPC cell motility by inducing an autocrine secretion of TGF-β1. In addition, flotillins have been reported to be involved in the initiation of signaling transduction through recruitment of receptor kinases, such as the Src tyrosine kinases (Src, Fyn, Lck), small GTPases and TNF-α receptor to lipid rafts, suggesting that FLOT1 may induce TGF-β signaling activation through recruitment of TGF-β receptors [17, 40, 41]. However, this hypothesis and whether FLOT1 overexpression can recruit TGF-β receptors to lipid rafts and induce signal transduction need to be further investigated.

In summary, this study reveals that elevated expression of FLOT1 plays an important role in lymph node metastasis in NPC and that FLOT1 is a critical activator of TGF-β signaling. Understanding the precise roles of FLOT1 in the pathogenesis and progression of NPC and activation of the TGF-β signaling pathway will increase our knowledge of the biological basis of cancer and may also enable the development of novel therapeutic strategies against NPC.

## MATERIALS AND METHODS

### Cells

The NPC cell lines SUNE1, CNE2, 5-8F, CNE1, HONE1 and 6-10B were established in Cancer Center of Sun Yat-sen University and maintained in RPMI 1640 medium (Invitrogen, Carlsbad, CA, USA) supplemented with 10% fetal bovine serum (HyClone, Logan, UT, USA), 100 U/ml penicillin and 100 μg/ml streptomycin (Invitrogen) in humidified atmosphere containing 5% CO_2_ at 37°C. Primary NPECs cultures were established as described previously [42] and grown in keratinocyte/serum-free medium (Invitrogen).

### Patient information and tissue specimens

Prior patient consent and approval from the Institute Research Ethics Committee were obtained for the use of all clinical materials for research purposes. Tumor tissue samples from a total of 169 patients with NPC were collected from the archives of the Department of Pathology of the Cancer Center, Sun Yat-sen University. The clinicopathological characteristics of the 169 patients are summarized in Supplementary Table 1. In addition, freshly-frozen tumor tissue biopsy samples were obtained from 22 patients with NPC who had (LN+, *n* = 14) or did not have (LN−, *n* = 8) lymph node metastasis, and two noncancerous nasopharyngeal biopsies were obtained under nasopharyngoscopy at the Department of Nasopharyngeal Carcinoma, Sun Yat-sen University Cancer Center.

### Immunohistochemistry

Immunohistochemical (IHC) analysis for FLOT1 was performed on the 169 formalin-fixed, paraffin-embedded NPC tissue sections as previously described [17]. The degree of immunostaining for each section was reviewed and scored separately by two independent pathologists. The scores were determined by combining the proportion of positively-stained tumor cells and the intensity of staining. Tumor cell proportions were scored as follows: 0, no positive tumor cells; 1, < 10% positive tumor cells; 2, 10%–35% positive tumor cells; 3, 35%–75% positive tumor cells; 4, > 75% positive tumor cells. Staining intensity was graded as: 1, no staining; 2, weak staining (light yellow); 3, moderate staining (yellow brown); 4, strong staining (brown). The staining index (SI) was calculated as the product of the staining intensity score and the proportion of positive tumor cells. Using this method of assessment, we evaluated protein expression in malignant lesions by determining the SI, with possible scores of 0, 2, 3, 4, 6, 8, 9, 12, and 16. Samples with a SI ≥ 8 were classified as high expression and samples with a SI < 8 were classified as low expression. Cutoff values were determined on the basis of a measure of heterogeneity using the log-rank test with respect to overall survival.

### Western blotting

Western blotting was performed as previously described [43], using anti-FLOT1 (Sigma, Saint Louis, MO, USA), anti-E-cadherin, anti-α-catenin, anti-N-cadherin, anti-vimentin, anti-p-Smad3 and anti-Smad3 (Cell Signaling, Danvers, MA, USA). The membranes were stripped and re-probed with anti-GAPDH (Sigma, Saint Louis, MO, USA) as a loading control.

### Vectors, retroviral infections and inhibitor

Human FLOT1 coding sequence was amplified by the polymerase chain reaction and subcloning into a pSin-EF2 vector. To silence endogenous FLOT1, 2 short hairpin RNA (shRNA) oligonucleotides were cloned into the pSuper-retro-puro vector to generate pSuper-retro-FLOT1-RNAi(s), respectively [17]. The TGF-β-responsive luciferase plasmid SBEx4 (Plasmid #16495) was purchased from Addgene (Cambridge, MA, USA); transfection efficiency was normalized by co-transfection of the cells with the pRL-TK plasmid (Promega, Madison, WI, USA). Retroviral production and infection were performed as described previously [17]. Stable cell lines expressing FLOT1 or FLOT1 shRNA were selected for 10 days with 0.5 μg/ml puromycin 48 h after infection. The shRNA sequences were: FLOT1-RNAi#1: CCCTCAATGTCAAGAGTGAAA; FLOT1-RNAi#2: ACAGAGAGATTACGAACTGAA; Smad3-RNAi: CTGTCCAATGTCAACCGGAAT. TGF-β inhibitor SB431542 was purchased from selleckchem (Houston, TX, USA), and dissolved in Dimethyl sulfoxide.

### Wound healing assay

Cell migratory ability was measured using the scratch assay. Briefly, cells were seeded into six-well plates in RPMI-1640 containing 10% FBS and cultured to form a confluent monolayer. Linear wounds were created in the cell monolayers using a sterile pipette tip. Images were captured and documented at 0 and 24 h after wounding using an inverted Olympus IX50 microscope with a 10× objective lens. Eight images per treatment were analyzed to determine the average position of the migrating cells at the edges of the wounds.

### Transwell matrix invasion assay

Cells (1 × 10^4^) were plated into the upper chamber of polycarbonate transwell filters coated with Matrigel (BD Biosciences, San Jose, CA, USA), cultured at 37°C for 24 h, then the cells inside the upper chamber were removed with cotton swabs and the cells that had migrated to the bottom surface of the membrane were fixed in 1% paraformaldehyde, stained with hematoxylin and counted in ten random fields of view per well.

### 3-D spheroid invasion assay

Cells (1 × 10^4^) were trypsinized, seeded in 24-well plates coated with 2% Matrigel (BD Biosciences), the medium was refreshed every other day and images of the cells were taken using a light microscope at 2-day intervals for 2 weeks.

### Inguinal lymph node metastasis model

The inguinal lymph node metastasis model was used to investigate the role of FLOT1 in lymph node metastasis in NPC as previously described [44]. Five week-old BALB/c nude mice (18–20 g) were purchased from the Center of Experimental Animal of Guangzhou University of Chinese Medicine (Guangzhou, China). All experimental procedures were approved by the Institutional Animal Care and Use Committee of Sun Yat-sen University and performed in accordance with the Declaration of Helsinki. Mice were randomly divided into eight groups (*n* = 8/group). CNE2-Vector vs. CNE2-FLOT1, CNE2-RNAi-vector vs. CNE2-FLOT1-RNAi, CNE2-FLOT1-RNAi-Vector vs. CNE2-FLOT1-Smad3-RNAi, and CNE2-FLOT1-RNAi-Vector vs. CNE2-FLOT1-FLOT2-RNAi (5 × 10^5^) were inoculated into the foot-pads of the mice on day 0. The mice were euthanized on day 28, and the primary tumors and inguinal lymph nodes were excised and paraffin embedded. Sections of the primary tumors and lymph nodes were subjected to H & E staining for histological examination. Metastatic tumor cells in the lymph nodes were stained with an anti-pan-cytokeratin antibody (ABcam, Cambridge, MA, USA) and evaluated by a pathologist. The images were captured using an AxioVision Rel.4.6 computerized image analysis system (Carl Zeiss Co. Ltd., Jena, Germany).

### Statistical analysis

All statistical analyses were carried out using SPSS 13.0 statistical software (SPSS Inc, Chicago, IL, USA). Comparisons between groups were performed with the Chi-square test. Univariate and multivariable survival analyses were performed using Cox regression analysis. Survival curves were plotted by the Kaplan–Meier method and compared using the log-rank test. In all cases, a *P*-value < 0.05 was considered statistically significant.

## SUPPLEMENTARY MATERIALS FIGURES AND TABLES


