# Supplementary material for: Upregulation of flotillin-1 promotes invasion and metastasis by activating TGF-β signaling in nasopharyngeal carcinoma
Source: Oncotarget. 2015 Dec 7;7(4):4252–64. doi: 10.18632/oncotarget.6483 (PMC4826203; doi:10.18632/oncotarget.6483)
Supplement: Supplementary file 1 [file oncotarget-07-4252-s001.pdf]

Upregulation of flotillin-1 promotes invasion and metastasis by activating TGF-β signaling in nasopharyngeal carcinoma

Supplementary Materials

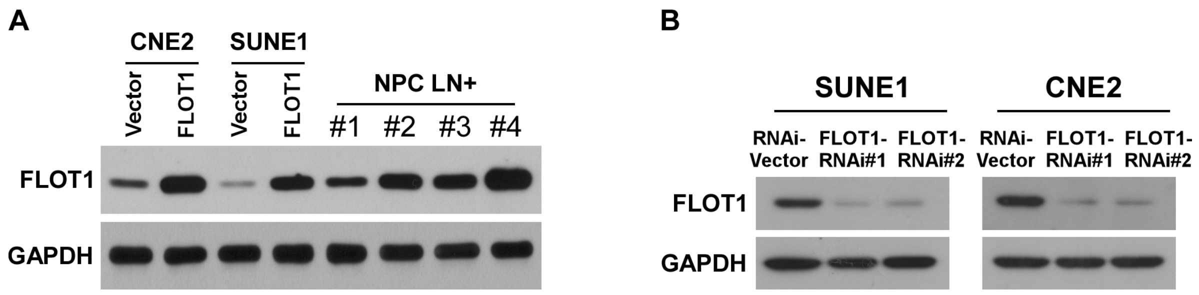

Supplementary Figure S1: Western blot analysis of FLOT1 in Vector control cells and FLOT1-transduced cells as well as 4 NPC LN+ tissues, or RNAi-vector control cells and FLOT1-silenced cells. GAPDH was used as a loading control.

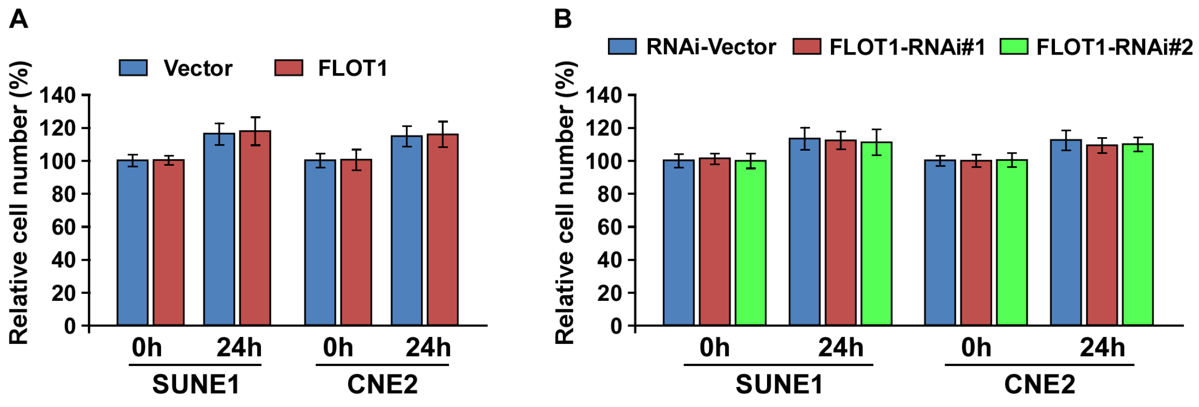

Supplementary Figure S2: The cell proliferation assay showed that neither FLOT1 overexpression (A) nor silencing (B) significantly change NPC cell numbers compared to controls within 24 h.

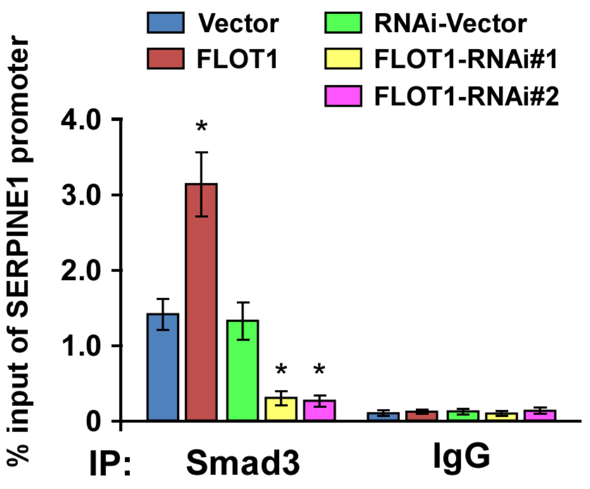

Supplementary Figure S3: ChIP assay revealed that the binding efficacy of Smad3 on SERPINE1 promoter was significantly increased in FLOT1-overexpressing CNE2 cells but decreased in FLOT1-silenced cells. *$P < 0.05$.

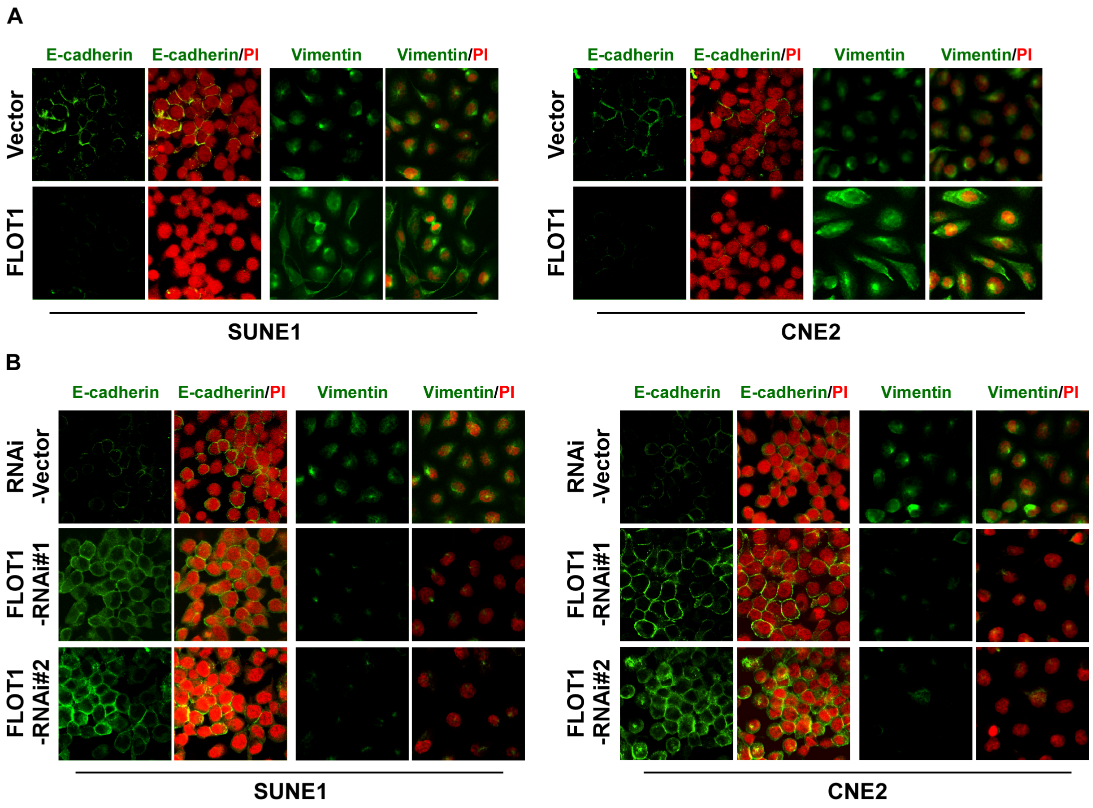

Supplementary Figure S4: FLOT1 overexpression facilitated, while silencing FLOT1 abrogated TGF-β-induced EMT in SUNE1 and CNE2 cells. (**A and B**) Fluorescent immunostaining of E-cadherin, Vimentin and Propidium iodide (PI) in the FLOT1-overexpressing (A) and FLOT1-silenced (B) NPC cells compared to controls. The green signal represents staining for E-cadherin or Vimentin, while the red signal signifies nuclear DNA staining with Propidium iodide.

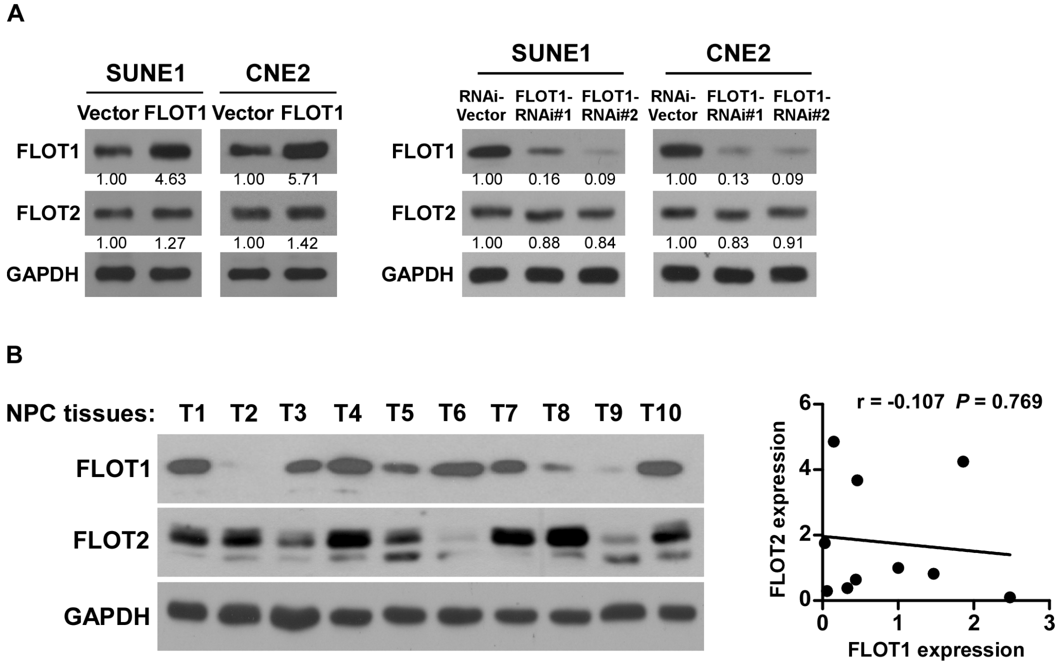

Supplementary Figure S5: (**A**) Western blotting analysis of FLOT2 in the FLOT1-overexpressing and FLOT1-silenced cells compared to controls. (**B**) Expression analysis (left) and correlation (right) of FLOT1 expression and FLOT2 protein expression in ten freshly collected human NPC tissue samples (T). The ratio of first sample T1 (FLOT1/GAPDH and FLOT2/GAPDH) was considered as 1.0. GAPDH was used as a loading control.

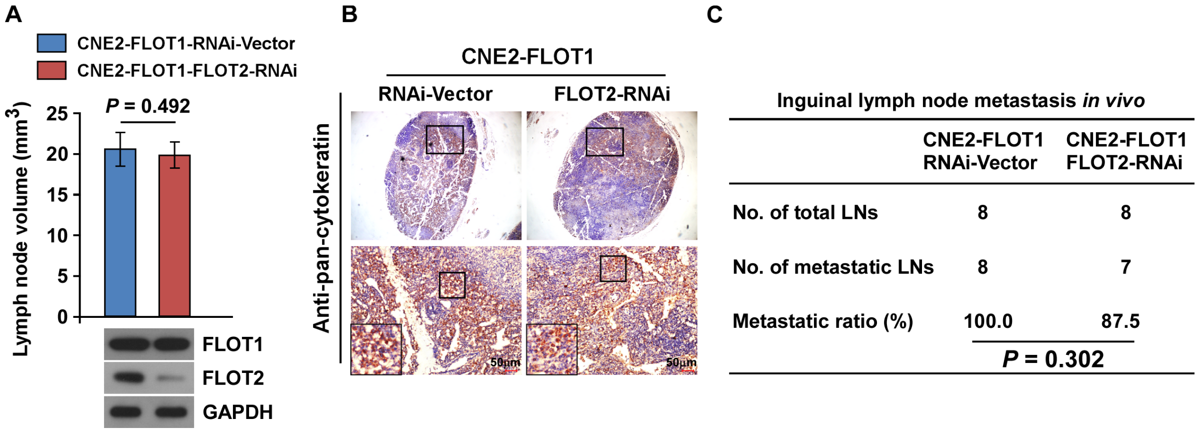

Supplementary Figure S6: FLOT1 promoted lymph node metastasis independent of FLOT2 in NPC. (**A**) The average volumes of inguinal lymph nodes in each group. (**B**) Representative micrographs of the inguinal lymph nodes immunostained with an anti-pan-cytokeratin antibody. Scale bars: 50 µm. (**C**) The ratios of metastatic to total dissected inguinal lymph nodes from mice inoculated with the indicated cells, the Chi-square test was used for statistical analysis. Error bars represent mean ± SD.

Supplementary Table S1: Clinicopathological characteristics of studied patients and expression of FLOT1 in NPC

Factor	NO.	(%)
**Gender**		
Male	126	74.6
Female	43	25.4
**Age (years)**		
≤ 45	86	50.9
> 45	83	49.1
**WHO histologic classification**		
NKUC	115	68.0
NKDC	51	30.2
KSCC	3	1.8
**Clinical stage**		
I	13	7.7
II	40	23.7
III	67	39.6
IVa-b	49	29.0
**N classification**		
N_0	57	33.7
N_1	72	42.6
N_2	32	18.9
N_3	8	4.8
**T classification**		
T_1	28	16.6
T_2	47	27.8
T_3	69	40.8
T_4	25	14.8
**VCA-IgA**		
< 1:80	26	15.4

≥ 1:80	143	84.6
**EA-IgA**		
< 1:10	40	23.7
≥ 1:10	129	76.3
**Recurrence or uncontrolled**		
NO	100	59.2
Yes	69	40.8
**Vital status**		
Alive	117	69.2
Dead	52	30.8
**Expression of FLOT1**		
Low expression	70	41.4
High expression	99	58.6

KSCC, keratinizing squamous cell carcinoma; NKDC, nonkeratinizing differentiated carcinoma; NKUC, nonkeratinizing undifferentiated carcinoma.

Supplementary Table S2: Univariate analysis of different prognostic parameters in patients with NPC

Factor	No. patients	Mean survival (months)	Hazard ratio	95% CI	*P*-Value
**Expression of FLOT1**					
Low expression	70	80.1	1.0	Reference	
High expression	99	62.8	2.631	1.403–4.933	0.003
**Gender**					
Male	126	69.5	1.0	Reference	
Female	43	73.3	0.755	0.388–1.469	0.407
**Age (years)**					
≤ 45	86	75.5	1.0	Reference	
> 45	83	65.5	1.778	1.020–3.099	0.042
**WHO histologic classification**					
NKDC/KSCC	54	71.8	1.0	Reference	
NKUC	115	70.2	1.126	0.624–2.029	0.694
**Clinical stage**					
I–II	53	79.2	1.0	Reference	
III–IV	116	66.9	2.164	1.076–4.277	0.030
**N classification**					
N_0	57	82.2	1.0	Reference	
$N_{1–3}$	112	65.2	2.771	1.350–5.686	0.005
**T classification**					
$T_{1–2}$	75	71.9	1.0	Reference	
$T_{3–4}$	94	69.8	1.101	0.635–1.910	0.731
**VCA-IgA**					
< 1:80	26	74.3	1.0	Reference	
≥ 1:80	143	69.7	1.481	0.632–3.470	0.366
**EA-IgA**					
< 1:10	40	74.5	1.0	Reference	
≥ 1:10	129	69.0	1.652	0.778–3.510	0.192

Supplementary Table S3: Multivariate analysis of different prognostic parameters in patients with NPC

Factor	Hazard ratio	95% CI	*P*-Value
**Expression of FLOT1**			
Low expression	1.0	Reference	
High expression	2.089	1.099–3.968	0.024
**Age (years)**			
≤ 45	1.0	Reference	
> 45	1.169	0.628–2.177	0.622
**Clinical stage**			
I–II	1.0	Reference	
III–IV	1.772	0.881–3.562	0.109
**N classification**			
N_0	1.0	Reference	
$N_{1–3}$	2.347	1.002–5.498	0.049
